# Do quality of life, anxiety, depression and acceptance improve after interdisciplinary pain rehabilitation? A multicentre matched control study of acceptance and commitment therapy-based versus cognitive–behavioural therapy-based programmes

**DOI:** 10.1177/03000605211027435

**Published:** 2021-07-18

**Authors:** Marcelo Rivano Fischer, Marie-Louise Schult, Monika Löfgren, Britt-Marie Stålnacke

**Affiliations:** 1 Rehabilitation Medicine, Health Sciences Department, Lund University, Lund, Sweden; 2 Department of Neurosurgery and Pain Rehabilitation, Skane University Hospital, Lund, Sweden; 3 Division of Rehabilitation Medicine, Department of Clinical Sciences, Danderyd University Hospital, Karolinska Institute, Stockholm, Sweden; 4 Department of Rehabilitation Medicine, Danderyd University Hospital, Stockholm, Sweden; 5 Department of Community Medicine and Rehabilitation, Rehabilitation Medicine, Umeå University, Umeå, Sweden

**Keywords:** Chronic pain, rehabilitation, pain acceptance, psychological intervention, multicentre study, interdisciplinary, cognitive–behavioural therapy, acceptance and commitment therapy, anxiety, depression

## Abstract

**Objective:**

Interdisciplinary pain rehabilitation (IPR) usually employs a cognitive–behavioural therapeutic (CBT) approach. However, there is growing support for chronic pain treatments based on acceptance and commitment therapy (ACT). Most studies of ACT and CBT for chronic pain have evaluated their effects after psychological interventions, not after IPR. We compared the results of an ACT-based IPR programme with two CBT-based IPR programmes.

**Methods:**

We used a retrospective multicentre pretest–posttest design with matched patient groups at three centres. Data were collected from the Swedish Quality Registry for Pain Rehabilitation before and after IPR participation. Participants completed the EQ-5D health-related quality of life questionnaire, the Chronic Pain Acceptance Questionnaire, (CPAQ) and the Hospital Anxiety and Depression Scale (HADS). Analyses were performed to compare the effects of the different interventions.

**Results:**

Neither EQ-5D nor HADS depression scores were affected by the psychological approach used. The score changes on both CPAQ subscales (activity engagement and pain willingness) indicated significant improvements between admission and discharge at all centres.

**Conclusions:**

These findings indicate the effectiveness of using psychological approaches to manage chronic pain. Both CBT and ACT had a beneficial effect on most of the assessed health-related parameters.

## Introduction

The often-cited definition of pain emphasizes that pain is always a subjective sensation related to emotional and psychological experiences.^1^ According to a European survey of chronic pain, approximately 20% of the Swedish population experiences chronic pain of moderate to severe intensity.^2^

Chronic pain is a complex condition that substantially affects many aspects of daily life, such as quality of life, working status, psychological well-being and self-esteem. In addition to causing individual distress, chronic pain has serious negative effects on society and national expenditure.^3,4^ However, there are individual differences in how pain severity and complexity interact with other symptoms and thus affect each individual’s activity levels and participation in society. Individuals who find it difficult to manage the effects of pain on daily living, work and leisure require approaches that address the complexity of the different aspects of chronic pain.

Interdisciplinary pain rehabilitation (IPR) programmes have been reported to show positive long-term results. Two systematic reviews found that IPR produces better results than less extensive treatment or no treatment at all,^5,6^ and the effect appears to be stable over time. IPR is based on a biopsychosocial model of chronic pain that includes somatic, psychological, environmental and personality aspects.^7–9^ The IPR approach comprises teamwork involving the participation of individuals from several professions; patients are usually also included as active team members. IPR interventions are synchronized and require structured, goal-directed plans. Most IPR programmes in Sweden use a cognitive–behavioural therapeutic (CBT) approach based on a combination of physical and psychological interventions. A 2015 Cochrane review of subacute neck pain treatments found low to moderate evidence for the effectiveness of CBT.^10^ However, there is growing support for the use of treatments based on acceptance and commitment therapy (ACT) for patients with chronic pain.^11^

ACT aims to improve function by increasing psychological flexibility. Studies have shown positive results after ACT treatment for disability, depression, anxiety and physical functioning.^12,13^ One systematic review (2017) found that ACT was more effective than controls in clinical settings (treatment as usual or no intervention).^13^ However, a review and meta-analysis by Ost (2014) found only a small and non-significant effect size of 0.16 when ACT was compared with different CBT treatments.^14^ Most studies of ACT and CBT for patients with chronic pain have examined the effects after psychological interventions, not after interventions conceptualized as IPR. Therefore, a multicentre study was designed to compare CBT-based IPR programmes with ACT-based IPR programmes. Three centres located at university hospitals in different geographical regions in Sweden participated in the study.

The study aim was to compare the results of an ACT-based IPR programme with two CBT-based IPR programmes using data from matched individuals registered in the Swedish Quality Registry for Pain Rehabilitation (SQRP). The research questions were a) Are there improvements in quality of life, level of pain acceptance, anxiety and depression after IPR? b) Do these improvements differ according to the approach used (ACT-based versus CBT-based IPR programmes)?

## Method

A retrospective multicentre pretest–posttest design with three matched patient groups was used. All patients participated in interdisciplinary rehabilitation programmes for long-term musculoskeletal pain. This study was performed in accordance with the Declaration of Helsinki, and the regional ethics review board approved the study (Dnr 2012/644-31/3). The study reporting conforms to the Strengthening the Reporting of Observational Studies in Epidemiology (STROBE) statement.^15^

Most specialized pain rehabilitation centres in Sweden report data to the SQRP. The effects of IPR have been continuously evaluated by the SQRP since 1998. The SQRP questionnaire includes items on sociodemographic factors (sex, age, education level and referral sources), pain duration, pain intensity and pain location, as described by Nyberg et al.^16^

### Power analysis

The power estimation was based on the EuroQOL health-related quality of life questionnaire (EQ-5D) index values. For a design with two repeated measurements, an average 0.2 point increase, a standard deviation of 0.3, a p-level of 5% and two-tailed tests, 40 patients in each group were needed to obtain an 80% chance of detecting differences between the two groups. This sample size ensured a good chance of detecting small-to-medium-sized differences between the groups.^17^

### Participants

After initial assessment, all participants completed a rehabilitation programme at one of the three interdisciplinary pain centres from January 2009 to December 2011. The patients were mostly referred from primary care or occupational health care. The centres are in the south (Centre 2), middle (Centre 1) and north (Centre 3) of Sweden. Inclusion criteria at the time of data gathering were (*i*) disabling chronic pain (on sick leave or experiencing chronic pain that interferes substantially with daily activities); (*ii*) aged 18 to 65 years; (*iii*) no further medical investigations needed; (*iv*) written consent for participation in the programme. Exclusion criteria were (*i*) ongoing major somatic or psychiatric disease; (*ii*) a history of major substance abuse or acute crisis.

### Matching

When estimating causal effects, the use of well-matched samples can reduce covariate bias. The goal is to replicate a randomized experiment as closely as possible by obtaining groups with similar covariate distributions. Matching methods for causal inference have been presented in a review by Stuart et al. and in recommendations by Gewandter et al.^18,19^

It is important to select appropriate covariates to include in the matching process. Matching variables were selected from those used in previous research on prediction of rehabilitation results and return to work.^19,20^ The selected covariates were also subclassified into (i) age categories (18–36, 37–45 and 46–67 years), (iii) country of birth (Sweden/Nordic countries vs. other countries), (iv) education level (primary school vs. secondary school/university level), (v) depression categories according to the Hospital Anxiety and Depression Scale (HADS) (<8, 8–10 and >10) and ratings of pain using a numeric rating scale (≤7 vs. >7). An SQRP statistician performed the matching procedure. The matching was performed by identifying suitable subjects comparable with the Centre 1 (middle region of Sweden) patients, who had participated in an IPR programme based on ACT. In contrast, the patients in Centres 2 and 3 (southern and northern regions) had participated in IPR programmes based on CBT. The SQRP database included 989 possible patients who ended their rehabilitation during the period January 2009 to December 2011. Of these, only 367 met the selection criteria; of the 367, a further 169 patients could not be matched to Centre 1 patients and were therefore excluded. This left 198 patients: 66 matched patients in each centre. Some of the patients in the 66 pairs identified had incomplete data (admission or discharge data); therefore, 44 pairs were identified who had high response rates on the SQRP questionnaire (EQ index score 93.2%–97.7%; EQ-visual analogue scale (VAS) score 95.5%–97.7%; Chronic Pain Acceptance Questionnaire (CPAQ) score 86.4%–97.7%); that is, 132 unique patients. The nearest neighbour matching procedure was used, ensuring the selection of individual patients with the smallest distance between them (i.e. high similarity). Each patient could only be used once to construct one pair (e.g. one patient in Centre 1 to one patient in Centre 2 or Centre 3, respectively).

### Data collection

Data were collected from the SQRP.^21^ Registry data are collected before and after participation in a rehabilitation programme. Because chronic pain is a complex condition that is affected by several interacting factors, instruments included in the SQRP questionnaire were used to measure health-related quality of life, acceptance of chronic pain, depression and anxiety. In the present study, analyses were performed on data from the EQ-5D, the CPAQ and the HADS.

#### EQ-5D

The EQ-5D is a standardized instrument that measures health-related quality of life.^22,23^ The EQ-5D is widely used to assess a variety of medical conditions and treatments. It provides a simple descriptive profile and a single index value for health status. The five EQ-5D dimensions are mobility, self-care, usual activities, pain/discomfort, and anxiety or depression. Each dimension has three levels (no problems, some problems, major problems) comprising 243 possible health states. The EQ-5D index score ranges from −1.0 to 1.0; a score of 1.0 indicates optimal health. A global self-rated health value is obtained using a VAS ranging from 0 (worst imaginable health state) to 100 (best imaginable health state).

The mean EQ-5D index score for the Swedish population aged 40 to 49 years is 0.86 (n = 2.870).^24^ The same study showed that people with neck/shoulder or low back pain had a lower EQ5D index score (0.66 to 0.70).

#### Chronic Pain Acceptance Questionnaire (CPAQ)

The CPAQ is a 20-item questionnaire designed to assess acceptance of chronic pain. The CPAQ contains 20 items that reflect activity engagement (doing activities in the presence of pain) and pain willingness (refraining from attempts to control or reduce pain).^25^ Each item is rated on a scale from 0 (never true) to 6 (always true); higher scores reflect greater acceptance of pain. The CPAQ has repeatedly demonstrated good psychometric properties.^11,26^ The psychometric properties of the Swedish version of the CPAQ used in this study are similar to those of the original English scale.^27^

#### HADS

The HADS is a 14-item questionnaire used to identify symptoms of anxiety and depression (7 items on the anxiety subscale and 7 items on the depression subscale) in hospital inpatients.^28^ The items are rated on a 4-point Likert scale (0–3). Both the original English version and the translated Swedish version of the scale have demonstrated satisfactory validity and reliability.^29^

#### IPR assessment and rehabilitation programmes

##### Assessment

The interdisciplinary pain team performed the interdisciplinary assessment at all centres using a biopsychosocial approach. Assessed patients were referred to the tertiary centres because of the complexity of their condition. Patients were individually interviewed and examined by each team member according to standardized protocols. The assessment day(s) led to recommendations, one of which was to undergo a rehabilitation programme at the centre.

There were some differences between the centres in length of the assessment period (1–3 days), the professions involved in the assessment team (All centres: physician, psychologist, physiotherapist and social worker; Centre 1: also occupational therapist) and the procedure used to discuss findings with patients (Centre 1: meeting between patient and physician after team meeting without patient; Centres 2 and 3: meeting between patient and team and other stakeholders).

##### The rehabilitation programmes

The programmes in all centres were implemented in groups consisting of 8 to 10 participants. Rehabilitation was based on interdisciplinary team collaboration, and the patient was considered an active team member. Several core sessions were conducted in group settings in all centres. The patients were also given individual activities and contacts according to their individual goals. Based on each patient’s needs and with the patient’s agreement, at the end of the programme contacts were made with external collaborating partners such as workplaces, the Swedish Social Insurance Agency and the patient’s general practitioner.

##### Dosage and intensity of the three IPR programmes and problem severity

The three centres were compared on variation in the number of days of rehabilitation, number of days per week and number of hours per day. Using previously proposed intensity categories,^30^ we determined that all centres provided a high-intensity IPR with more than 100 hours of rehabilitation. In a 2020 study, Rivano-Fischer et al.^31^ investigated differences in the severity of patients’ problems between university hospital units and other units in all regions in Sweden. They found that patients in university hospital centres had significantly lower EQ5D index scores than those in medium units (p = 0.004) (small effect size [Cohen’s d = 0.33]) and lower EQ-VAS (health status) scores than those in large units (p < 0.001) (small effect size [Cohen’s d = 0.18]). The three centres used in the present study are all at the university hospital level.

##### The ACT-based rehabilitation programme at Centre 1

The rehabilitation programme was standardized according to ACT principles^32^ and a manual^33^ was used as a framework for the interventions. The focus was on building up a behavioural repertoire in valued life directions, rather than emphasizing reductions in pain and distress. Weekly, the rehabilitation team emphasized one of the dimensions according to the manual (the present moment, values, committed actions, self-as-context, defusion and acceptance).^31^ Typically, two team members (e.g. a psychologist and a physiotherapist) worked together. This model has been successfully used in team-based pain rehabilitation.^34–36^ The team received ongoing tutoring from the ACT manual during the first 2 years. The 8-week rehabilitation programme was considered high-intensity IPR (>100 hours) and was mostly group-based, with the potential for individual adaptations during specific sessions and individual sessions if needed. The programme included physical training, body awareness training, exposure training, activity-based exposure and training, activity balance training, creativity groups and group therapy. The programme was provided on 3 days/week for 6 hours/day.

##### The CBT-based rehabilitation programmes at Centres 2 and 3

The outpatient rehabilitation programmes at Centres 2 (4 weeks) and 3 (5 weeks) were based on cognitive–behavioural principles and focused on pain management, education about pain and its consequences, and bodily and psychological reactions to pain. The programmes included physical and occupational therapy, information and training on coping strategies, information about bodily and psychological reactions to chronic pain, relaxation and body awareness training. Other sessions included in the programme addressed ergonomics and education about pain mechanisms and their consequences. Except for the programme duration and number of participants, the only differences between the IPR structure in Centres 2 and 3 were that Centre 2 exhibited a stronger tendency toward group-based interventions than Centre 3 and that contacts with other stakeholders in Centre 2 were made based on individual needs.^37^ The programmes were provided 5 days/week, 6 to 7 hours/day, and were considered high-intensity IPR programmes (>100 hours).

### Data analysis

Data were analysed using SPSS Statistics for Windows, Version 17.0. (SPSS Inc., Chicago, IL, USA). Non-parametric statistical methods were used because all data were measured using ordinal scales and the sample was small. Basic descriptive statistics were also calculated to determine whether there was a significant difference between the three rehabilitation settings (Friedman test). Any significant differences found were examined using Wilcoxon’s signed rank test to test for differences between Centre 1 and Centre 2 and between Centre 1 and Centre 3. Changes within each centre’s pre- and post-rehabilitation programme were investigated using the Wilcoxon signed rank test.

## Results

### Scores at baseline

Demographic information (age, sex, education level, and country of birth) for patients in all centres is shown in Table 1.

**Table 1.** Demographic information for participants from the three centres.

Centre	1	2	3	Total
Total number of pairs	n = 44	n = 44	n = 44	n = 132
Age groups (years)				
18–36	10	10	10	30
37–45	18	18	18	54
46–67	16	16	16	48
Sex				
Female	39	39	39	117
Male	5	5	5	15
Education level				
Other		3		3
Primary school	7	5	3	15
High school	25	19	27	71
University	12	16	14	42
No information		1		1
Country of origin				
Sweden and other Nordic countries	42	42	42	126
Europe and outside Europe	2	2	2	6

There were significant differences at baseline between Centres 1, 2 and 3 on EQ index score; subjects in Centre 1 tended to score lower than subjects in Centres 2 and 3 (Wilcoxon signed rank test: n = 41, p = 0.002 between Centres 1 and 2; n = 43, p < 0.001 between Centres 1 and 3). There were also significant differences at baseline between Centres 1 and 2 on HADS depression score (Wilcoxon signed rank test: n = 38, p = 0.006); subjects in Centre 1 scored lower than subjects in Centre 2. There were significant differences between Centres 1 and 3 on CPAQ pain willingness (Wilcoxon signed rank test: n = 36, p = 0.049) and HADS anxiety score (Wilcoxon signed rank test: n = 39 p = 0.004); subjects in Centre 1 tended to score lower than subjects in Centre 3 (p = 0.053) (Table 2).

**Table 2.** Difference in baseline scores across centres for EQ-5D index, EQ-5D VAS, CPAQ (activity engagement and pain willingness) and HADS anxiety and depression.

	EQ-5D index	EQ-5D VAS	CPAQ 1	CPAQ 2	HADS depression	HADS anxiety
Pairs in analysis (n)	41	42	37	40	38	37
Centre 1–Centre 2 p-value	**0.002**	0.689	0.723	0.796	**0.006**	0.094
Pairs in analysis (n)	43	43	38	36	39	39
Centre 1–Centre 3 p-value	**<0.001**	0.110	0.450	**0.049**	0.053	**0.004**

CPAQ, Chronic Pain Acceptance Questionnaire; CPAQ 1, activity engagement; CPAQ 2, pain willingness; EQ-5D, EuroQOL health-related quality of life questionnaire; HADS, Hospital Anxiety and Depression Scale; VAS, visual analogue scale.

### Description of pre- and post-rehabilitation scores in each centre

Table 3 shows the pre- and post-rehabilitation scores on the EQ-5D index, EQ-5D VAS, CPAQ and HADS with minimum and maximum scores and percentiles for each centre.

**Table 3.** Pre- and post-rehabilitation scores, minimum and maximum values and percentiles for each centre on EQ-5D index, EQ-5D VAS, CPAQ (activity engagement and pain willingness) and HADS anxiety and depression.

			N		Min	Max	Percentiles		
	Centre		Valid	Missing			25	50 (median)	75
EQ-5D index	1	Pre	43	1	−0.239	0.760	−0.016	0.088	0.170
		Post	43	1	−0.239	0.796	0.030	0.099	0.689
	2	Pre	42	2	−0.239	0.760	0.088	0.158	0.656
		Post	43	1	−0.126	0.796	0.088	0.293	0.656
	3	Pre	43	1	−0.077	0.760	0.088	0.193	0.689
		Post	44	0	−0.239	0.760	0.160	0.620	0.689
									
EQ-5D VAS	1	Pre	43	1	6	80	25	30	50
		Post	43	1	1	90	25	45	66
	2	Pre	43	1	7	75	30	30	50
		Post	43	1	10	90	30	50	70
	3	Pre	44	0	10	75	30	42	60
		Post	43	1	10	80	30	45	65
									
CPAQ 1	1	Pre	42	2	7.0	47.0	15.0	21.0	29.3
		Post	43	1	2.0	61.0	24.0	32.0	39.0
CPAQ 2		Pre	43	1	5.0	37.0	15.0	20.0	25.0
		Post	43	1	7.0	48.0	21.0	27.0	34.0
									
CPAQ 1	2	Pre	39	5	3.0	41.0	15.0	22.0	27.0
		Post	41	3	10.0	46.0	20.0	24.0	32.0
CPAQ 2		Pre	41	3	4.0	36.0	14.0	19.0	26.0
		Post	40	4	5.0	31.0	16.3	22.5	26.8
									
CPAQ 1	3	Pre	39	5	6.0	46.0	18.0	24.0	34.0
		Post	41	3	8.0	53.0	22.5	31.0	36.0
CPAQ 2		Pre	37	7	4.0	42.0	19.5	25.0	32.0
		Post	41	3	11.0	44.0	19.5	26.0	29.0
HADS									
Depression	1	Pre	39	5	3.0	19.0	6.0	11.0	14.0
		Post	44	0	0.0	20.0	4.0	7.5	10.8
Anxiety	1	Pre	39	5	1.0	21.0	7.0	10.0	13.0
		Post	44	0	1.0	20.0	4.0	7.0	11.0
									
Depression	2	Pre	43	1	1.0	18.0	7.0	11.0	13.0
		Post	43	1	1.0	16.0	4.0	8.0	11.0
Anxiety	2	Pre	42	2	0.0	20.0	5.8	9.0	12.0
		Post	43	1	0.0	17.0	5.0	7.0	11.0
									
Depression	3	Pre	44	0	1.0	19.0	7.0	11.0	13.8
		Post	44	0	1.0	20.0	4.3	6.5	11.0
Anxiety	3	Pre	44	0	1.0	19.0	4.0	7.0	11.0
		Post	44	0	2.0	19.0	4.0	7.0	8.0

CPAQ, Chronic Pain Acceptance Questionnaire; EQ-5D, EuroQOL health-related quality of life questionnaire; HADS, Hospital Anxiety and Depression Scale; VAS, visual analogue scale.

### Within-group changes after the rehabilitation programme

The Centre 1 participants had improved significantly at discharge on all assessments; that is, they experienced greater acceptance of pain (doing activities despite the presence of pain [p < 0.001] and refraining from attempts to control or reduce pain [p < 0.001]), better reported health status (p = 0.039), better quality of life (p = 0.034) and less anxiety (p < 0.001) and depression (p < 0.001). Almost the same improvements were reported for Centre 2, except for EQ-5D index score, which did not significantly improve. Improvements were also noted in Centre 3, whose patients increased their activity engagement (p = 0.001) and had lower depression at discharge (p < 0.001) (Table 4).

**Table 4.** Changes for each centre on EQ-5D index, EQ-5D VAS, CPAQ (activity engagement and pain willingness) and HADS anxiety and depression.

	Centre 1		Centre 2		Centre 3	
	n	p-value	n	p-value	n	p-value
EQ-5D index score	43	**0.034**	41	0.587	43	0.158
EQ-5D VAS	43	**0.039**	42	**0.02**	43	0.321
CPAQ 1	42	**<0.001**	38	**0.036**	36	**0.001**
CPAQ 2	43	**<0.001**	38	**0.048**	35	0.486
						
HADS depression	39	**<0.001**	43	**0.003**	44	**<0.001**
HADS anxiety	39	**<0.001**	42	**0.025**	44	0.062

CPAQ, Chronic Pain Acceptance Questionnaire; EQ-5D, EuroQOL health-related quality of life questionnaire; HADS, Hospital Anxiety and Depression Scale; VAS, visual analogue scale.

### Between-group changes from pre- to post-rehabilitation

Health-related quality of life and depression level were not affected by the type of rehabilitation programme. However, there was a significant difference in anxiety score changes between admission and discharge between Centres 1 and 3 (p = 0.039) (Table 5). There were significant differences in the score changes between admission and discharge between Centre 1 (ACT-based rehabilitation programme) and Centres 2 and 3 (CBT-based rehabilitation programmes) on the CPAQ subscales activity engagement (Centre 1 vs. Centre 2: p = 0.004; Centre 1 vs. Centre 3: p = 0.006) and pain willingness (Centre 1 vs. Centre 2: p < 0.001; Centre 1 vs. Centre 3: p < 0.001) (Table 5).

**Table 5.** Comparison of score changes between centres on EQ-5D index, EQ-5D VAS, CPAQ (activity engagement and pain willingness) and HADS anxiety and depression.

	EQ-5D index score	EQ-5D VAS	CPAQ 1	CPAQ 2	HADS depression	HADS anxiety
Pairs in analysis (n)	40	41	36	37	38	39
**Centre 1–Centre 2 p-value**	0.096	0.878	**0.004**	**<0.001**	0.121	0.321
Pairs in analysis (n)	42	42	35	34	37	39
**Centre 1–Centre 3 p-value**	0.126	0.341	**0.006**	**<0.001**	0.260	**0.039**

CPAQ, Chronic Pain Acceptance Questionnaire; EQ-5D, EuroQOL health-related quality of life questionnaire; HADS, Hospital Anxiety and Depression Scale; VAS, visual analogue scale.

## Discussion

The present study, which used matched comparison groups, showed that IPR with both ACT and CBT interventions improved important health-related parameters in patients with chronic pain. The main difference between the psychological approaches used as a basis for IPR was in the scores on the CPAQ subscales activity engagement and pain willingness. Only patients from Centre 1, who received the ACT-based IPR, reported improvements on both subscales, suggesting that IPR based on ACT methodology increases pain acceptance more than CBT-based IPR.

The baseline data suggested that patients in Centre 1 were admitted to IPR with poorer health-related quality of life and experienced greater depression and anxiety than patients in Centres 2 and 3. However, patients in Centres 1 and 2 improved on almost all the included variables, and patients from Centre 3 showed improvements on two variables. These results suggest that IPR based on both the ACT and CBT methodologies produces substantial improvements on several important health-related areas, although some differences may exist.

A recent meta-analytic review of acceptance and mindfulness-based interventions found small effects for pain intensity, depression, disability and quality of life. However, a moderate effect was found for anxiety and pain interference.^38^ Even though CBT has been the leading psychological treatment for patients with chronic pain, only small to medium effect sizes have been found for CBT interventions compared with controls.^39^ Wetherell et al. (2011) found few differences between ACT and CBT, although a few measures showed small effects in favour of CBT.^36^

The present findings demonstrated that different psychological-based IPR programmes had a positive effect on several important areas of health. Targeted areas such as pain acceptance showed greater improvement after the ACT-based programmes.

Centre 1 used a protocol-driven ACT intervention at the time of observation, whereas Centres 2 and 3 followed the CBT chronic pain model. However, because research in ACT methodology is developing progressively, the possibility cannot be ruled out that Centres 2 and 3 incorporated some ACT-related principles into their interventions, at varying degrees, at the time of data collection; Centres 2 and 3 did not use any ACT manual, training or supervision at the time of this study.

An important aspect of using matching methods is to determine the quality of the resulting matched samples. All matching should be followed by an assessment of the covariate balance in the matched groups, where balance is defined as the similarity of the empirical distributions of the covariates in the matched groups. In the present study, the three matched groups were considered to be balanced. However, the matching procedure was fraught with difficulties. For example, more patients in Centre 3 were of higher education level, and fewer were immigrants, than patients in Centres 1 and 2. The matching procedure started with a large number (989) of patient participants in the IPR programmes and resulted in 132 suitable patients (44 pairs), which indicates a problem with the design used. This may be because the patients participating in the ACT programme served as a basis for identifying eligible pairs; we searched for complete patient data and used several inclusion criteria. We reduced bias by not using multiple controls for each patient at Centre 1.

To summarize, this study demonstrates the effectiveness of psychological approaches for the management of chronic pain. Both CBT and ACT are effective in improving important health-related parameters in patients with chronic pain. However (and not unexpectedly), ACT seems more effective in increasing acceptance of chronic pain, a concept that is foundational to ACT but not to CBT.

### Limitations

To compare ACT-based versus CBT-based IPR, patients at three centres were matched on five key variables that are confounding factors for pain rehabilitation outcomes. These variables were age, education level, country of birth, level of depression/anxiety and perceived pain intensity levels. However, although the matching procedure was carefully conducted, an important confounding variable may have been missed and some variability may not have been controlled for. Additionally, the lack of randomization may have reduced the internal validity (making it more difficult to differentiate the rehabilitation programmes in terms of their effectiveness).

After matching, the sample size was rather small, which may have affected the reliability of the cause-and-effect relationship found. Non-parametric statistical methods were used because of the small sample size.

Furthermore, the three centres did not follow one unified protocol (except for choosing between ACT and CBT); thus, other variables may have affected the results. However, although the treatment duration varied between the three centres, they shared important characteristics that ensured that the IPR delivery was generally similar: all provided high-intensity IPR programmes (>100 hours), were accredited by the Commission of Accreditation of Rehabilitation Facilities,^40^ were university clinics and followed the SQRP regulations on pain rehabilitation. The study was conducted in centres in the southern, central and northern parts of Sweden, which helped to avoid sociogeographical biases. However, we cannot rule out the possibility that some regional differences affected the results.

## Conclusions

This study demonstrated the effectiveness of psychological approaches in the management of chronic pain and their effect on important health-related parameters. Both CBT and ACT had a beneficial effect on most of the parameters measured.
